# Anxiety-like behaviour is attenuated by gabapentin, morphine and diazepam in a rodent model of HIV anti-retroviral-associated neuropathic pain

**DOI:** 10.1016/j.neulet.2008.10.005

**Published:** 2008-12-19

**Authors:** Victoria C.J. Wallace, Andrew R. Segerdahl, Julie Blackbeard, Timothy Pheby, Andrew S.C. Rice

**Affiliations:** Pain Research Group, Department of Anaesthetics, Pain Medicine and Intensive Care, Faculty of Medicine, Imperial College London, Chelsea and Westminster Hospital Campus, 369 Fulham Road, London SW10 9NH, UK

**Keywords:** Neuropathic pain, Pain comorbidity, Open field activity, HIV neuropathy, Gabapentin

## Abstract

Neuropathic pain is commonly associated with affective disorders such as anxiety and depression. We have previously characterised a rodent model of HIV, anti-retroviral-associated neuropathy in which rats develop hypersensitivity to a punctate mechanical stimulus and display anxiety-like behaviour in the open field paradigm. To assess the potential of this behavioural paradigm for the assessment of pain related co-morbidities in rodent models of pain, here we test the sensitivity of this anxiety-like behaviour to the analgesic agents gabapentin and morphine in comparison to the known anxiolytic drug diazepam. We found that gabapentin (30 mg/kg, i.p.) and morphine (2.5 mg/kg, i.p.), which reduce mechanical hypersensitivity in these rats, significantly reduces measures of thigmotaxis in the open field. The effect of gabapentin and morphine did not differ significantly from diazepam (1 mg/kg, i.p.). This study highlights the potential use of this rodent model and behavioural paradigm in the validation of the affective component of novel analgesic pharmacological targets and elucidation of underlying pathophysiological mechanisms.

Chronic neuropathic pain arises as a direct consequence of a lesion or disease affecting the somatosensory system [34]. For example, direct nerve trauma; diseases such as diabetes and HIV; and therapies including anti-retroviral- and chemo-therapy are sometimes associated with spontaneous pain and occasionally evoked pain associated with signs of sensory gain such as hyperalgesia or allodynia. Moreover, there is significant co-morbidity between neuropathic pain and neuropsychiatric disorders, including anxiety and depression [5,26,33]. The high prevalence and deleterious impact of affective illness in chronic pain sufferers is well recognised [12,22,30,36] yet there is little evidence to support common pathophysiological mechanisms [22,35].

Rodent models have proven valuable in identifying pathophysiological mechanisms and therapeutic targets for emotional disorders [4]. Extensive research has characterised the behavioural phenotypes of an array of rodent models of neuropathic pain [6,20,32,39]. However, the majority of such studies rely on measures of evoked withdrawal responses to identify hypersensitivity states and therefore reveal little, if any, information regarding emotional or subjective aspects of pain that are so commonly observed in the clinic and measured in clinical trials [9]. Recently however, a number of studies have begun to tackle this problem by measuring pain and anxiety-like behaviour in rodent models of neuropathic pain; combining hind paw withdrawal tests with anxiety paradigms such as the open field and elevated plus maze [15,16,23,29,38,37,40]. These data suggest that such behavioural paradigms are useful for the assessment of pain related co-morbidities in rodent models and may therefore prove useful in the elucidation of underlying pathophysiological mechanisms and validate potential pharmacological analgesic targets.

We have previously characterised a rodent model of anti-retroviral-associated neuropathy in which rats develop hypersensitivity to a punctate mechanical stimulus and display anxiety-like behaviour in the open field paradigm [38]. Gabapentin and morphine are effective analgesic agents in human neuropathic pain conditions [10,17], and are known to reduce mechanical hypersensitivity in various models of neuropathic pain [15,38,37]. The current study assesses the effect of these analgesic agents on the anxiety-like behaviour in anti-retroviral-treated rats in comparison to the known anxiolytic drug diazepam. This study aims to draw comparisons between the anti-hypersensitivity/analgesic and anxiolytic properties of these drugs in an effort to correlate pain and co-morbidity behaviour in models of neuropathic pain. Correlations between such behavioural traits would highlight the use of such models and behavioural measures in future research.

All experiments conformed to the British Home Office Regulations and IASP guidelines [41]. Male Wistar rats weighing 200–250 g were used for all experiments (B&K, Hull, UK) and were housed in a temperature-controlled environment, maintained on a 14:10 h light–dark cycle (experiments were performed during the light phase) and provided with feed and water *ad libitum*.

Animals were injected intraperitoneally with 0.5 ml of ddC solution (50 mg/kg in sterile saline; Sigma–Aldrich, Poole, UK) or as a control, sterile saline three times a week for up to 3 weeks (Monday, Wednesday, Friday) [38,40].

Testing of hind paw reflex behaviour was performed as described previously [15,38,37,40]. Briefly, the threshold for hind paw withdrawal in response to punctate mechanical stimulation was measured in conscious animals using an electronic “von Frey” device [27] of 0.5 mm^2^ probe tip area (Somedic Sales AB, Sweden).

Baseline measurements were obtained for all rats over the course of a week prior to start of ddC treatment. Hind paw reflex thresholds were then assessed by a “blinded” observer. The threshold value at each time point tested was calculated as the mean ± S.E.M.

Between days 19 and 21 following the first ddC injection; rats with established hind paw hypersensitivity to punctate mechanical stimulation (a change of at least 30% from baseline) were randomised into treatment group and administered intraperitoneally with either; gabapentin (Pfizer Ltd.; 30 mg/kg in saline), morphine sulphate (Sigma–Aldrich 2.5 mg/kg, in saline) [doses as previously described [15,38,37]], diazepam [Sigma–Aldrich; 1 mg/kg [31] in 1:2 mix of ethanol (absolute molecular grade; VWR, Poole) and cremophor EL (Univar; Essex, UK)] or vehicle (sterile saline or ethanol/cremaphor). Each animal was only used once for this experiment. 40 min post-injection, rats were placed into a 100 cm × 100 cm arena illuminated to 4 lx with a defined inner zone of 40 cm × 40 cm. Locomotion of the rats within the arena was tracked over 15 min using a Sanyo VCB 3372 high-resolution monochrome camera (Tracksys, Notts, UK) and stored and analysed with Ethovision software v.3 (Tracksys) as described previously [15,38,37]. The total distance moved, time spent in the inner zone and the number of entries into the inner zone were calculated and displayed as the mean ± S.E.M. For all experiments, the experimenter was “blinded” to drug treatments received.

Sigmastat version 2.03 (SPSS Inc., Surrey, UK) was used to determine the presence of statistically significant differences (*minimum p* ≤ 0.05) throughout the study. All groups were compared using a Kruskall–Wallis one-way analysis of variance (ANOVA) with Dunn's all pairwise or multiple comparisons vs. control *post hoc* analysis.

In line with previous studies [38], rats treated with ddC developed a significant (*p* ≤ 0.05) hypersensitivity to punctate mechanical stimulation by days 19–21 (33.2 ± 1.9 g) as compared to baseline (49.3 ± 1.9 g) or sham controls (48.6 ± 2.3 g) (Fig. 1). Rats that had developed at least 30% change from baseline (*n* = 60; eight excluded) were then randomised into groups for the open field test.

At the time of peak mechanical hypersensitivity, ddC-treated rats (*n* = 12) receiving no injection prior to the open field test showed no significant difference in the total distance moved (6461 ± 542 cm) vs. sham control rats (7503 ± 568 cm) (Fig. 2A), suggesting a lack of overt motor deficit. However, ddC-treated rats displayed a significantly (*p* ≤ 0.01) reduced number of entries into the inner zone (3.9 ± 0.8 vs. 10 ± 2.0) (Fig. 2B) and time spent in the inner zone (10.7 ± 2.1 s vs. 15.9 ± 1.6 s) (Fig. 2C) as compared to sham control animals (*n* = 9). This is characteristic of thigmotaxis, thought to be associated with anxiety-like behaviour.

To test the sensitivity of the thigmotactic behaviour to a standard anxiolytic drug, we assessed the effect of diazepam on thigmotactic behaviour in ddC-treated rats. When administered 40 min prior to testing, diazepam (*n* = 9; 1 mg/kg, i.p.) significantly (*p* ≤ 0.05) increased the number of entries into (6.2 ± 0.5 vs. control; 2.5 ± 0.3) (Fig. 2B) and time spent in the inner zone (18.0 ± 1.7 vs. control; 9.0 ± 1.4 s) (Fig. 2C) of the open field as compared to vehicle control treatment.

We have previously demonstrated that gabapentin (30 mg/kg, i.p.) and morphine (2.5 mg/kg, i.p.) significantly reverse mechanical hypersensitivity displayed by ddC-treated rats [38]. Therefore to assess the association between hypersensitivity and anxiety behaviour in ddC-treated rats we assessed the effect of these drugs on thigmotactic behaviour in the open field. Gabapentin (*n* = 9; 30 mg/kg, i.p.) and morphine (*n* = 11; 2.5 mg/kg, i.p.) significantly (*p* ≤ 0.05) increased the number of entries into the inner zone (gabapentin: 6.1 ± 0.7; morphine: 8.2 ± 1.7; control: 2.5 ± 0.3) (Fig. 2B) and time spent in the inner zone (gabapentin: 22 ± 3.9 s; morphine: 23.9 ± 5.4 s; control: 9.0 ± 1.4 s) (Fig. 2C) of the open field arena as compared to vehicle control treatment. The effect of gabapentin and morphine was not significantly different from diazepam for either parameter. Furthermore, none of the drugs significantly altered the total distance moved (gabapentin: 6896 ± 563; morphine: 6702 ± 418; diazepam: 5086 ± 716; control: 5099 ± 318 cm) (Fig. 2A), suggesting a lack of overt motor effects by any treatment. As there was no significant difference between values for saline vehicle control (*n* = 10) or cremaphor/ethanol vehicle control (*n* = 9) prior to open field testing, the data for these groups has been pooled.

Neuropathic pain is commonly associated with affective disorders such as anxiety and depression [5,26,33]. Previous studies have demonstrated measures of anxiety-like behaviour in rodent models of neuropathic pain [15,38,37], yet few have determined if the hypersensitivity indicative of a pain state is actually correlated with the anxiety behaviour observed. Here we have demonstrated that rats treated with the antiretroviral ddC display behaviour associated with mechanical hypersensitivity and anxiety both of which can be targeted by the analgesic agents, gabapentin and morphine.

The open field paradigm is based upon the rodent's conflict between an innate aversion to exposed spaces and a tendency to explore novel environments [4]. Reduced exploratory behaviour characterised by thigmotactic (wall-hugging) behaviour is classically interpreted as anxiety-like behaviour. In support of the hypothesis that thigmotactic behaviour in our rat model of antiretroviral related neuropathy is anxiety-like, we have demonstrated it's susceptibility to the benzodiazepine class of anxiolytic; diazepam. Of interest, a recent study [29] demonstrated that anxiety-like behaviour displayed by neuropathic rats in an alternative behavioural paradigm, the elevated plus maze, was normalised by the benzodiazepine, midazolam, without any analgesic effect. Although slightly different drugs with respect to receptor targets, this suggests that diazepam is unlikely to be acting as an analgesic in this study but rather as an anxiolytic.

Gabapentin has been shown to be efficacious in reducing measures of pain-like behaviour in rodent models of pain [1,7,8] and is a known analgesic in human neuropathic pain conditions [10,17], including HIV-neuropathy in the short term [14]. Morphine attenuates hypersensitivity in rodent neuropathy models [2,21] and evidence is accumulating to suggest that opioids relieve symptoms of human neuropathic pain of peripheral origin [10,17]. We have previously demonstrated that gabapentin and morphine reverse hypersensitivity in ddC-treated rats [38] suggesting that the hypersensitivity displayed in this model may prove as a useful measure for testing the clinical utility of novel analgesic agents in the future. Our present data demonstrates that gabapentin and morphine also reverse thigmotactic behaviour. Together with the effect of diazepam, these data suggests that the thigmotactic behaviour observed in ddC-treated rats is indicative of an anxiety-like state that is associated with the pain state. In line with this, a previous study by Roeska et al. [29] demonstrated that gabapentin and morphine, at the doses used in this study, reverse anxiety-like behaviour in rodent neuropathic pain models without such effect in sham animals. Therefore, it would seem that in non-neuropathic rats, gabapentin and morphine drugs do not possess anxiolytic properties at these doses. Furthermore, in spite of previous evidence in which morphine reduces motility in rats in similar behavioural paradigms, in this study and the study by Roeska et al. [29], no negative effect of morphine on motility was observed. These data show that the effect of morphine is variable depending on the dose, animal model and route of administration employed. Our data therefore supports our hypothesis that the anxiety-behaviour measured in this study is associated with the pain state of the animal and is susceptible to analgesic compounds. Evidence suggests that different rodent behavioural tests, especially of anxiety, are likely qualitatively different from one another and may well model different aspects of the human conditions [3,4,18]. Therefore, in light of our results and the study by Roeska et al. [29], we feel that it will be useful to extend these data to other measures of anxiety- and also depression-related behaviours.

Until now, very little preclinical research in rodents has been able to test much more than basic measures of hypersensitivity. In contrast, clinical studies encompass a greater range of measures including subjective, qualitative and psychological components [24,25,28] which reflect the true extent of symptoms of chronic pain. Therefore, to draw closer parallels with the clinical scenario, there is a great need for a larger variety of tests preclinically. In such, further utilisation of clinically relevant rodent models such as those recently characterised to investigate mechanisms of varicella-zoster virus-associated neuropathy [11,13,15], HIV-neuropathy [38,37] and as in this case, drug induced neuropathy [19,38] in combination with integrative behavioural measures such as the open field paradigm will undoubtedly prove useful for the elucidation of mechanisms of chronic pain and co-morbidities and ultimately efficacious therapeutic targets.

## Figures and Tables

**Fig. 1:**
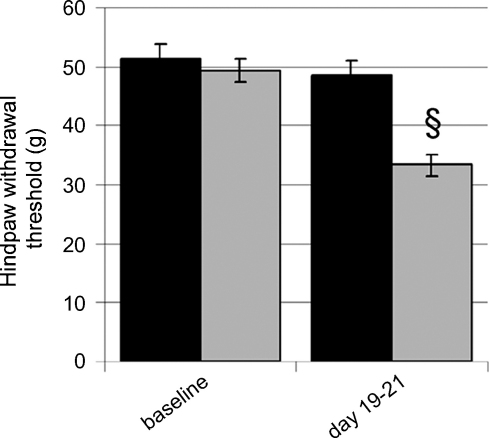
The development of hind paw reflex sensitivity to punctate mechanical stimuli in rats treated with ddC. Hind paw withdrawal thresholds to an electronic von Frey device measured following treatment with 50 mg/kg ddC (*n* = 60) or saline sham control (■ *n* = 10). Data is shown at baseline and at time of peak hypersensitivity (days 19–21). Statistical significance of differences between groups (^§^*p* ≤ 0.05) was determined by a Kruskall–Wallis one-way analysis of variance (ANOVA) with Dunn's all pairwise multiple comparisons *post hoc* analysis where. Each value is the mean ± S.E.M.

**Fig. 2:**
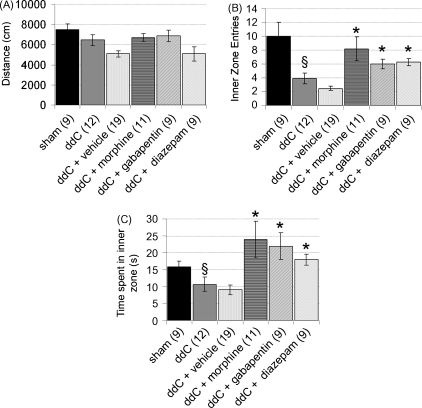
Gabapentin, morphine and diazepam attenuate thigmotactic behaviour of ddC-treated rats in an open field arena. (A) The total distance moved within the open field arena (1 m × 1 m) was assessed over 15 min and is not significantly altered in ddC-treated rats as compared to saline controls or in rats treated with drug or vehicle control 40 min prior to the open field test (number of rats in each group shown in brackets). (B) The number of entries into the inner zone (40 cm × 40 cm) and (C) time spent in the inner zone of the open field arena was significantly reduced in ddC-treated rats vs. sham controls. This reduction was significantly attenuated by gabapentin (30 mg/kg), morphine (2.5 mg/kg) and diazepam (1 mg/kg) when administered 40 min prior to testing. The statistical significance of differences between ddC treated and sham animals (^§^*p* ≤ 0.01) or drug treatment groups and the vehicle control group (^*^p ≤ 0.05) was determined using a Kruskall–Wallis one-way analysis of variance (ANOVA) with Dunn's multiple comparisons vs. control *post hoc* analysis. As the ddC + vehicle groups did not differ significantly from each other, this data was pooled for graphical display. Each value is the mean ± S.E.M.
